# Supplementary material for: Cell-Type-Specific Quantification of a Scaffold-Based 3D Liver Co-Culture
Source: Methods Protoc. 2019 Dec 23;3(1):1. doi: 10.3390/mps3010001 (PMC7189675; doi:10.3390/mps3010001)
Supplement: Supplementary file 1 [file mps-03-00001-s001.pdf]

**Supplementary Materials:**

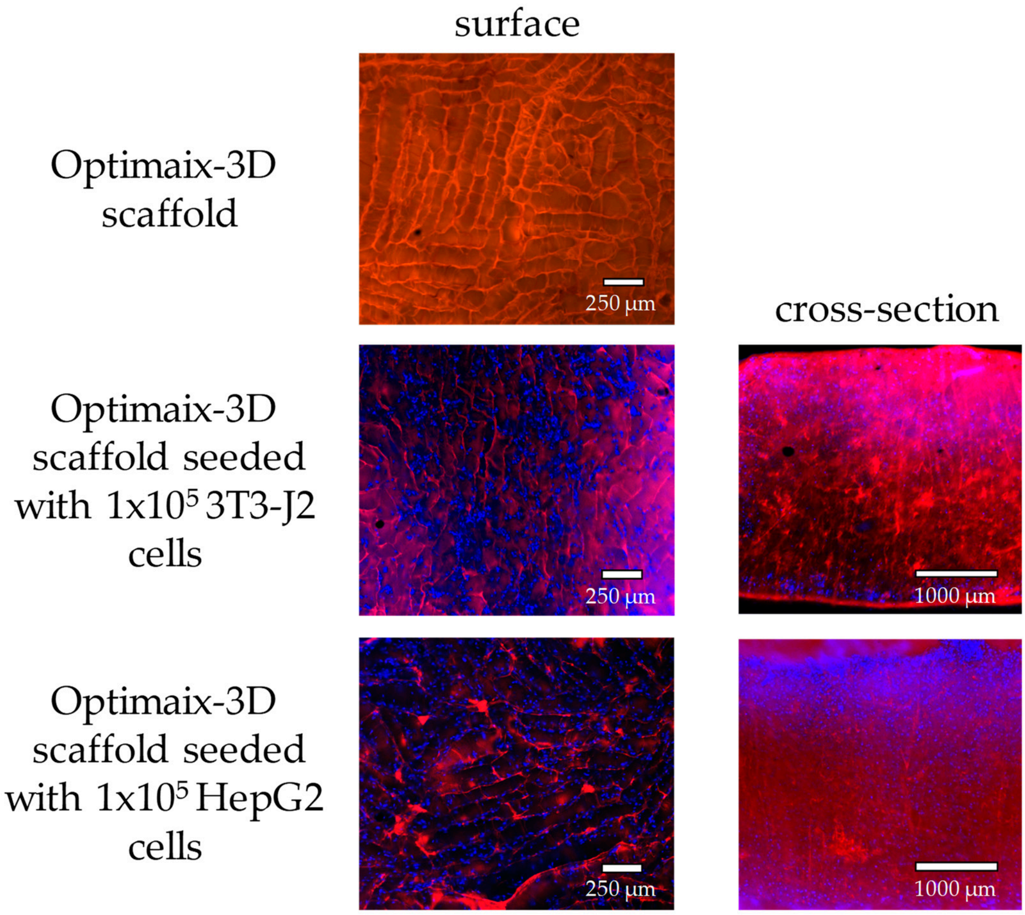


**Figure S1.** Representative pictures of the Optimaix-3D scaffold. The scaffold matrix was stained with SRB, the nuclei of seeded 3T3-J2/ HepG2 cells were stained with HOECHST 33342. Pictures of the scaffold surface are shown in 40-fold magnification, the cross-section pictures were shown in 20-fold magnification.

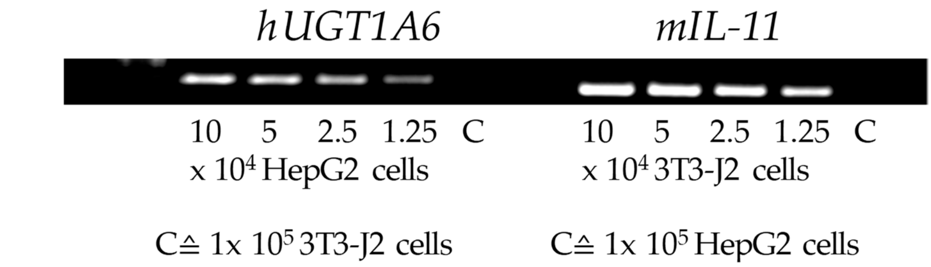


**Figure S2.** Optimization of the hUGT1A6 and the mIL-11 primer, PCRs were optimized to ensure that the PCR products are in the logarithmic phase. Additionally, species- specificity of the used primers was guaranteed by testing the highest concentration of the respective other cell type.

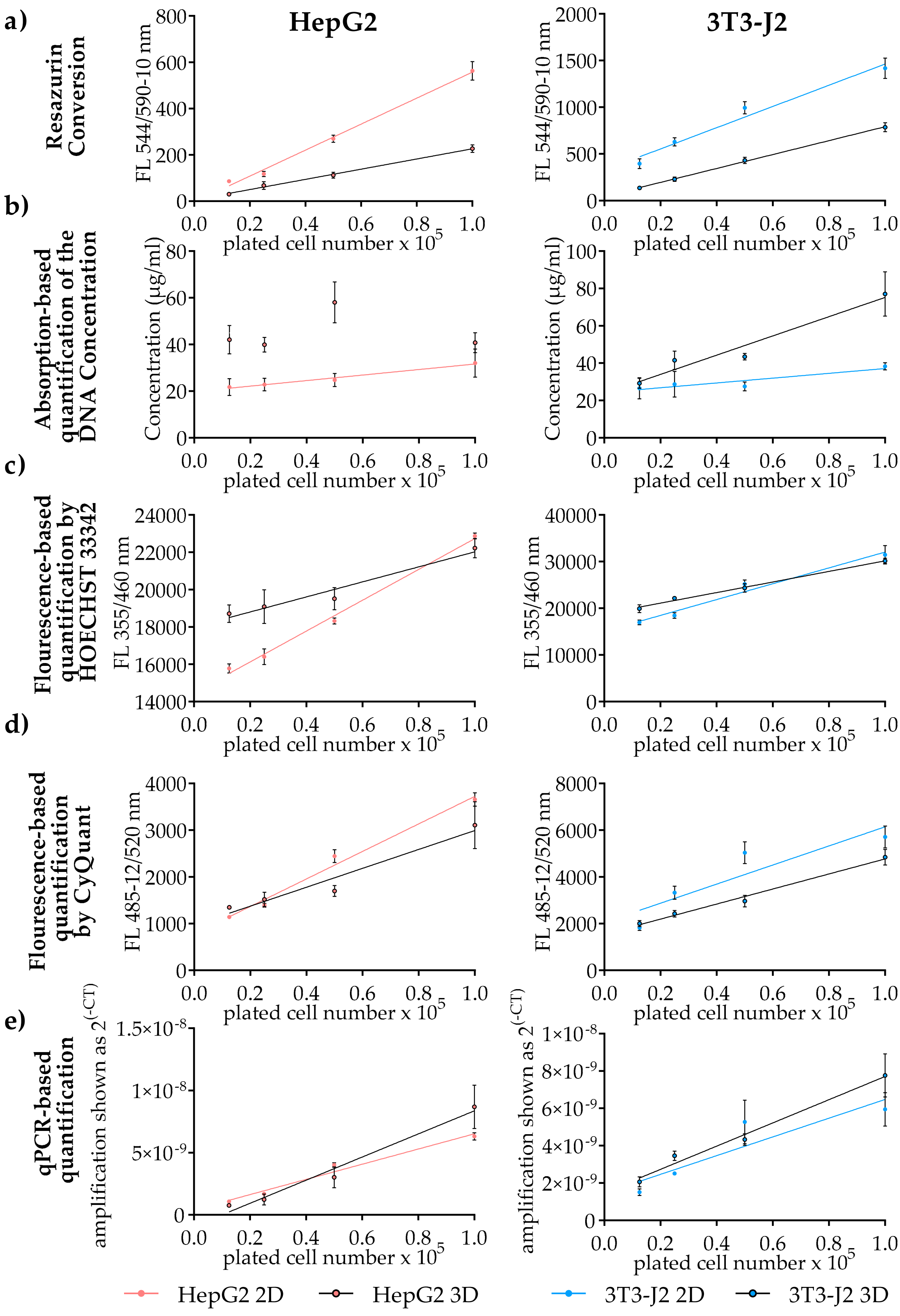


**Figure S3.** Comparison of different approaches for 2D and 3D scaffold cell culture quantification. a) Results of resazurin conversion, b) Absorption-based DNA quantification, c+d) Fluorescence-based DNA measurement using Hoechst 33342 and CyQuant, and e) Quantification of absolute cell numbers by qPCR with cell-type specific primers. HepG2 and 3T3-J2 cells were plated as mono-cultures in 2D and 3D using different cell numbers. Data measured and the corresponding linear regressions are shown separated by cell type for each quantification technique. N = 3, n = 2; mean ± SEM.

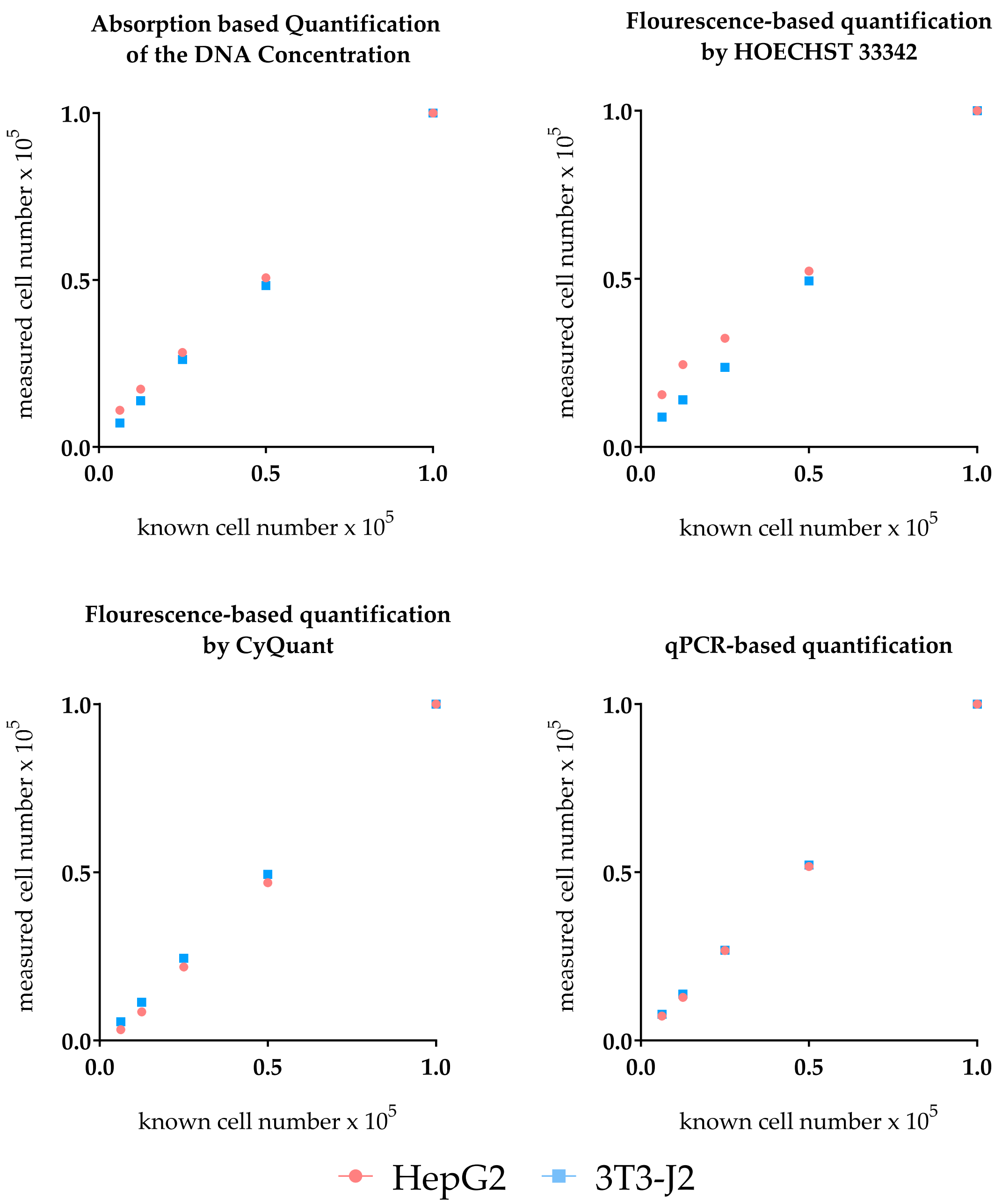


**Figure S4.** Standard curves of the different DNA based approaches used for calculation of the limit of detection, limit of quantitation and the sensitivity of each method.

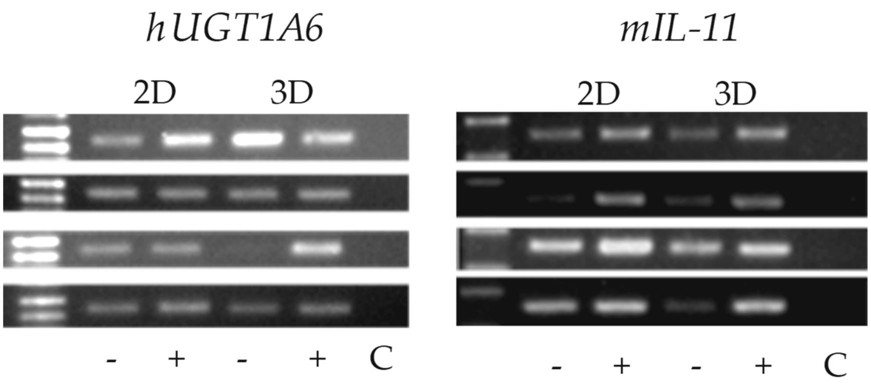


**Figure S5.** Gel pictures of all four runs of the co-culture experiment analyzed via conventional PCR showing co-cultures [+], monocultures [-], and additionally a negative control (c) consiting of DNA from the 2D monoculture of the other cell line. The pUC19/Msp I marker was used for *hUGT1A6* and for *mIL-11* the Bioline Hyperladder II was used.
